# A new perspective into molten corium concrete interaction for interpreting Fukushima Dai-Ichi terrace-shaped debris

**DOI:** 10.1038/s41598-025-09107-7

**Published:** 2025-07-02

**Authors:** A. P. Pshenichnikov

**Affiliations:** Collaborative Laboratories for Advanced Decommissioning Science, Fukushima Research and Engineering Institute, Japan Atomic Energy Agency, Tomioka, Fukushima Japan

**Keywords:** Nuclear waste, Volcanology

## Abstract

This paper compares open-source volcanic lava images with official TEPCO videos and reports. The fuel debris formation at Fukushima Dai-Ichi Nuclear Power Station (1F) Unit 1 shows similarities to natural lava flows at various volcanoes, including submarine eruptions and pillow lava. Based on evidence found in 1F debris images, the author suggests the unconventional interaction between molten corium and concrete (MCCI). The MCCI could have started in upper pedestal region due to an RPV failure above the RPV skirt, which is higher than usually considered. Additional volume, which cannot be explained now, was explained in the study as mass of MCCI products created in the upper areas of pedestal where hot corium first reacted with concrete. This unconventional MCCI would have brought enough melt to reach the observed terrace-shaped debris height. The current paper attempts to establish the fact that the natural mechanism of 1F terrace-shaped debris formation is easily identifiable through a multidisciplinary approach, which opens the scientific communities of volcanologists and materials scientists to a collaboration.

## Introduction

**Fig. 1:**
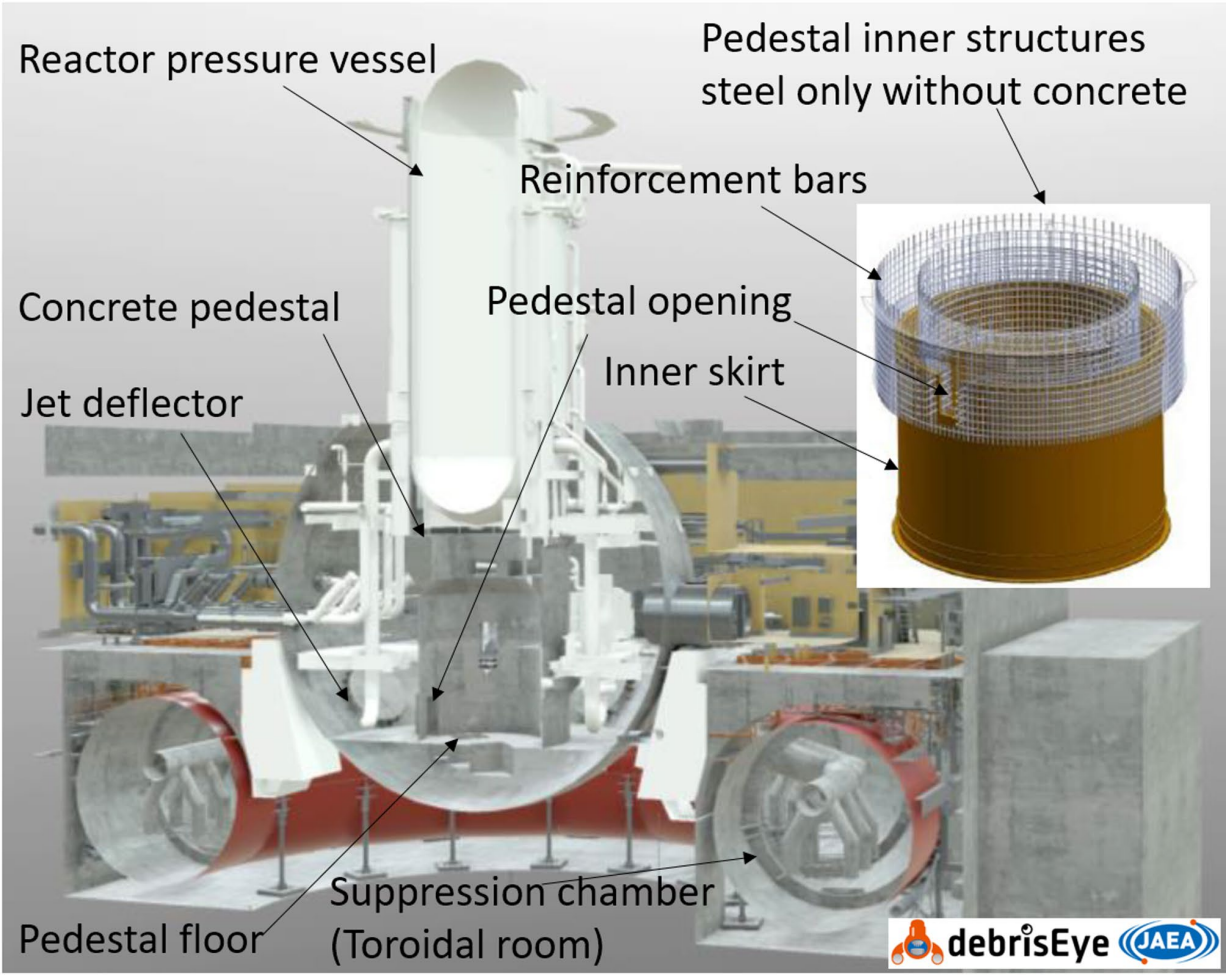
Scheme of the Unit 1 developed using 3D view application debrisEye adapted from Yamashita, et al.^4^.

 During the inspection of the debris inside the primary containment vessel (PCV) of the Fukushima Dai-Ichi Nuclear Power Station (1F)^1,2^ (Fig. 1), it was found that the concrete wall was ablated to the height of approximately 1.3 m above the initial floor level around the circumference inside the Unit 1 pedestal^3^. The floor and the wall below the fuel debris level could also be damaged, but it cannot be seen under the pile of molten and then solidified debris. While damage was concentrated mostly inside of the pedestal, particularly strong ablation was observed near the pedestal entrance. A 1.2 m-thick concrete wall disappeared completely inside and outside of the pedestal, leaving only the reinforcement steel bars (rebars) and the massive metallic structure, which is called the inner skirt (Fig. 1).

According to the recent estimation^3^the debris volume exceeds that which could be produced if all the core materials are melted and relocated to the pedestal. Outside of the pedestal, right above the area where concrete had disappeared, a 30 mm-thick solid shell remained in a shape of a bookshelf or a terrace^3^ (Fig. 2). A significant amount of material beneath the crust had disappeared. It was assumed that it remained liquid under the crust, which allowed further relocation in the course of the accident. An analogy with natural lava flows and formation of lava tubes with crust accumulation above is coming to mind immediately after looking at the consequences of the accident. However, hot corium in the RPV vessel melts at significantly higher temperatures.

Degradation of concrete by reaction with corium is a well-known phenomenon in the severe accident community. Those not familiar with this subject are highly advised to refer to the latest state-of-the-art report on molten corium – concrete interaction during a severe accident published under the auspices of the International project of the Organisation for Economic Co-Operation and Development (OECD) Nuclear Energy Agency (NEA)^5^.

The crust pattern that was observed in the 1F Unit 1 looks similar to those crusts formed after several MCCI tests in the past reported in some ANL MCCI tests such as MACE M1B^6^ or CCI-2^5^. However, the most similar appearance of crust is considered to be the that formed as a result of the MCCI tests COMET-L2 and -L3^7^. The test conditions guaranteed the fact that material beneath the crust was liquid leaving well distinguishable temperature degraded zone and a kind of icicles under the crust. However, the absence of rebars in the tests does not allow explaining why the concrete was so perfectly cleaned away from the steel reinforcement bars^3^. Tests with reinforced concrete like MOCKA tests were having initial temperature of melt in the range of 1750–1900°C^5^. In those tests, all rebars melted together with concrete. Unfortunately, there were no test, which could fully explain the behaviour of the 1F Unit 1 debris and concrete degradation in the pedestal floor. Thus, up to date there is no consensus reached regarding the phenomena responsible for the terrace-shaped debris and the melt withdrawal with survived rebars.

There are several versions of the accident progression currently under discussion in the severe accident community, which are critically reviewed in this paper. Even though the problem of wall degradation by melting is well known and clarified in the tests, the question of survived reinforcement steel and anchored crust formation together with further propagation of molten debris is not well investigated. Anchored crust was observed in the MCCI tests before, but never in combination with rebars as most tests use various kinds of electrical heating that prevents inclusion of metallic rebars. In volcanology anchored crust formation is probably out-of-interest, though happens in the form of cracked lava pillows and lava tubes.

Recently, interdisciplinary approach was applied for studying corium flow using equations developed for volcanic lava. It became possible by joint efforts of severe accident experts together with volcanologists^8^. Can knowledge in volcanology help in the interpreting of the 1F Unit 1 severe accident progression and consequences too?

The problem of formation of the terrace-like crust structure continues to excite the minds of materials scientists and severe accident specialists. The Fig. 2 had even become the cover image of the biennial International Workshop on Fukushima Dai-Ichi Decommissioning Research FDR2024 (fdr2024.org). One of the main questions is how this terrace was formed? How and why the molten material broke the crust and continued propagation? Currently, it seems there is no plausible answer on the mechanism of the terrace formation for the case of the 1F Unit 1. This paper attempts to clarify this question.

## Critical literature review

To date, several hypotheses were proposed about the hollow crust formation at the pedestal bottom of the 1F.

Because terrace-like deposits were anchored higher than the final debris position, a volume increase due to foaming of the crust at the top of the MCCI products was proposed as potential mechanism. It was explained by Tourniaire for a series of experiments in the past^9^. However, the bubbles in those studies were artificially introduced into various concrete melt compositions. Among silicate lava materials, none could have so many gas-forming elements that can produce so many bubbles as artificial bubbling did. Perhaps it would make more sense if 1F concrete was at least partly made of limestone, since limestone thermal degradation causes generation of large amount of CO_2_ gas^10,11^. But analysis of the 1F concrete bore sample proved that it is basalt concrete^12^ thus limestone concentration is negligible. Or perhaps concrete could degas quickly at a temperature around 2000–2500 °C. However, steel bars survived, which automatically limits the temperature about 1400 °C. At this temperature, degassing of silicate concrete melt proceeds slowly more like a cooking porridge. In addition, foam is definitely not a solid crust, thus if the underlying material disappears, foam will likely to adapt to the new level, and the formation of a terrace would be impossible. Moreover, foaming cannot be kept constant in the whole volume of concrete. For example, near the hottest area it should be more bubbles produced than on the periphery. Thus foaming crust should be thinner in areas far from the hot centre. Considering viscosity of silicate lava, it would prevent fast relocation of bubbles from the centre of bubbling to the periphery. In addition, the surface would probably be much more irregular if caused by foam. Hence, the foaming concept cannot be applied for the 1F crust formation near the pedestal opening and cannot explain crust thickness anchored at the concrete wall.

Two other versions were proposed as the most probable during the 32nd Nuclear Regulation Authority meeting (2022) of the 1F accident analysis study group^13^. The assumed process for the Unit 1 was that liquefied (U, Zr)O_2_ debris melted through the RPV and then relocated to the pedestal bottom as a large pile of debris to start MCCI. According this version, crust would form after the MCCI product has formed. However, it seems that crust should form much earlier due to high solidus temperature of (U, Zr)O_2_. The crust would form even before MCCI actually proceeds. If the crust we see on the images is entirely made of (U, Zr)O_2_, beneath the crust there should be also liquid (U, Zr)O_2_ melt, which covers the rebar. Corium liquidus temperature would be far above steel melting temperature and probably the steel bars would not be able to survive same as in the MOCKA test (see in the OECD NEA report^5^). However, steel bars remained clean and look not much degraded in the Fig. 2, which implied the temperature below 1400 °C. This fact makes silicate nature of lava the first priority just because (U, Zr)O_2_ based material would not be liquid by such temperature. Thus, melt of solely (U, Zr)O_2_ also doesn’t explain the debris we had observed in the Unit 1 PCV. One should not completely rebut this version; because a part of it may be true, in particular, the crust UO_2_ content maybe higher than the further MCCI products. However, it can be confirmed only by sampling and detailed analysis, which was not performed for the crust.

The second hypothesis was based on the following. It was experimentally shown that hydrothermal reactions occur at around 200 °C at high pressure. Concrete components (especially SiO_2_) dissolve in high-temperature, high-pressure water to form water-containing glass. At 200 °C for keeping the water in liquid phase, it implies a PCV pressure over 15 bars. However, the maximum design pressure of the 1F Unit 1 PCV is only 5 bars. Such high overpressure would never be achieved in Unit 1 PCV. Thus, water could never be condensed at 200 °C in PCV. Moreover, crust was supposed to form over the layer of liquid water, which implies flatness of the crust. It seems inapplicable because TEPCO had measured gradual decrease in the level of the debris crust^3,14^. The level was not flat, thus, such structure could not be formed at a water surface.

One of the most straight-forward and undoubtedly the most realistic, is the hypothesis of concrete ablation by melting at the pedestal bottom. It can be derived from the results of various research groups worked with molten corium – concrete interaction phenomenon over years^5^. Indeed, in the 1F Unit 1, a typical MCCI pattern is evident: the distinct markings remained at the wall higher than the final position of the pedestal debris, remaining material has an appearance as it was molten and then had solidified, extensive concrete wall ablation^3^. A terrace-like crust had formed on the surface of debris when they relocated to the pedestal bottom. Relicts of it stay anchored at the position 200–300 mm higher than the final debris level. The total height could decrease by gradual spreading outside of the pedestal and ablation of concrete floor and walls. The abrupt ablation was not confirmed in the tests^15^. Thus, ablation is assumed to gradually occur after relocation and first solidification of top crust.

How much concrete loses in volume upon melting? There are many ways how concrete reduces the volume when becomes molten. First moisture evaporation, then chemically bound water evaporation like decomposition of portlandite, then CaCO_3_ decomposition and CO_2_ evaporation, air from porosity evaporation, then degassing of concrete agglomerates and in addition to all of this, SiO_2_ is relatively volatile at high temperatures on its own. For typical siliceous and basalt concretes, formation of CO_2_ is not negligible, but incomparably low in comparison to a limestone concrete. The majority of the volume reduction in case of siliceous concrete would result from water evaporation. Typically, quartzite or basalt concrete may lose at least 20% of its volume when it completely melts into glass^16^. For CaCO_3_ limestone concretes this number maybe double as high^10^. However, basaltic and siliceous concretes emit Si-bearing aerosol, which also should not be neglected. In the ACE program impact of metallic Zr on decomposition of liquefied concrete and generation of aerosols on typical types of concretes during MCCI was studied^17^. The most interesting comparison with 1F Unit 1 is possible, particularly using the results of ACE-L2 and ACE-L6 tests. Both had siliceous concrete (69 mass % of SiO_2_). The main difference was in initial oxidized/metallic Zr ratio in the melt, being 70/30 and 30/70 mole % for L2 and L6 respectively. The highest generation of aerosols in the off-gas was detected for the test L6 with 70% metallic Zr^17^. If metallic Zr content of 1F Unit 1 melt is higher than expected after severe oxidation in steam during RPV melt relocation, it is going to contribute to additional aerosol generation (i.e. pedestal debris volume decrease).

The thickness of crust depends on the balance between cooling rate, heat transfer and flow conditions, defined by viscosity of lava. The crust thickness in COMET-L2 MCCI test varied 20–50 mm. The crust thickness in 1F Unit 1 was about 30 mm. Usually, in VULCANO tests, crusts would raft on the surface follow the entire mass of the hot lava during spreading^11^. Though the vertical walls in VULCANO were made of refractory ceramics, one can argue that it is different from the behaviour of a concrete wall. However, similar floating usually happens in natural volcanic lava flows. The best way to form a thick crust is anchoring and cooling under minimal spreading. If the crust is fixing some cool obstacle, still liquid material inside can decrease level, while the crust remains anchored. In absence of spreading, the greatest ablation should occur in the highest heat flux areas. According to Bouyer, et al.^15^, for 1F it should be the bottom direction because it is expected due to the properties of the 1F basaltic concrete. Is it true or not, cannot be confirmed now. Based on many photos and video evidences one can confirm that the final height of debris in the pedestal inner part was higher and in the pedestal opening visually 3–4 times smaller. Such uneven volume decrease seems impossible by only concrete floor melting. No one would exclude floor melting process out of consideration completely, but spreading of the MCCI products near the opening could have played an important role as well. Having discussed this, it becomes clear, that currently observed molten material volume reduction near pedestal opening is doubtful to occur simply by melting of the concrete floor. To reduce the height by 200 mm, one has to melt 1000 mm of concrete. According to TEPCO^3^ the crust position and final debris height differ by 90 cm. If this were only by floor melting, the concrete floor near pedestal area should have melted to a depth of 4.5 m below the initial floor level. However, even now it is problematic to explain the currently high volume of debris.

To summarize, there is a lack of understanding on where the additional material came from? Why do we observe the hollow crust? Where has material disappeared? Why do we observe the cleaned rebars? These are the questions, which should be answered.

If melting of the floor to such depth is not realistic, there can be additional reasons, for example, spreading of MCCI product out into the outside pedestal area. Because 1F Unit 1 is a BWR type with Mark-I containment, it can be compared with American design. MCCI product spreading scenario was considered for the Mark-I containment of an American type of BWR reactor long before the 1F accident^18^. However, to realize spreading scenario, does MCCI lava product have to brake the crust, which has already been formed?

At the time of writing the paper, it seems like there is no flawless theory, which described the phenomena of lava and crust formation inside of the 1F Unit 1. However, this paper provides a different perspective and suggests a process of lava propagation and terrace crust formation in the 1F Unit 1.

## Results: a comparison of the observed 1F unit 1 debris and natural volcanic lavas

To better understand the behaviour of molten material, let us look first at the types of phenomena that are common in nature. The Figs. 2, 3, 4, 5, 6, 7 were combined into the Table 1, where the left-hand side showing the phenomena observed in the Unit 1 PCV and the right-hand side showing images of natural lava flows under subaerial and submarine conditions. Let us compare them and see if the 1F situation could be analogous to natural lava behaviour. Perhaps an explanation from natural sciences can be found for the formation of the 1F Unit 1 terrace crust.

In the Fig. 2 material beneath the crust had disappeared, but the metal structures were intact. In fact, when natural lava flows over a metal fence, the metal structure usually survives above the lava (Fig. 3). According to the USGS (United States Geological Survey) data^19^ the temperature of Kīlauea eruption is usually about 1170 °C. The lava tubes have about 1250 °C. It seems very similar to siliceous concrete melting temperatures, thus the behaviour of lava can be easily extrapolated to molten concrete behaviour in the 1F Unit 1 pedestal. The metallic fence survived when liquid lava attacked it, same performed the rebars of the 1F when molten concrete attacked them. However, if it was liquid corium mixture with steel from the RPV, its temperature is sufficiently higher and as it was shown in the MOCKA test and other tests^20^ rebar would be melted together with concrete. The crust once solidified around the metallic or another massive cool object, would be fixed in that position. Another similar feature, shown in both images is the smooth surface of the lava, which is only possible when lava moves slowly under subaerial condition^21,22^. Thus, similarity between volcanic lava flow and 1F Unit 1 pedestal debris becomes obvious.

 In the Fig. 4 the terrace-like crust was found in the pedestal opening. To explain this behaviour let us look at the Fig. 5, where the same style of crust was created in nature.

In the Fig. 5, submarine pillow lava suddenly burst causing the liquid material flowing out. After bursting, the crust had rapidly stabilized by water cooling at around + 4 °C from both sides. Cooling seems to be the key factor for stabilizing the hollow crust. The Figs. 4 and 5 have many similar features, thus it is likely that the lava and 1F silicate lava debris had similar formation mechanism. One cannot predict the exact temperature conditions, but the mechanism of crust formation looks same. To limit the temperature range of MCCI lava in the 1F pedestal, let us start from the fact that the rebars survived, which means that temperature range is limited from above at about 1400 °C. Siliceous concrete does not melt congruently. However, major volume fraction the concrete becomes liquid at about 1200–1250 °C, which limits the range from below. Typical lava tubes crust forms when lava temperature is around 1250 °C, which slightly increases the lower limit. Thus, the author suggests that the temperature of the MCCI product flowed through the pedestal opening was in the range of 1250–1400 °C.

Volcanic lava crust breaks because of natural events occurring in parallel to the lava extrusion. It can be either internal pressure pushing from the main magma channel, aftershock earthquakes, and thermal cracking due to contraction, and gravity influence on the preliminarily cracked surface. All of these could influence the 1F crust too. In addition to these, such events as pedestal flooding after the accident and possibility that a fallen object could break the crust cannot be excluded.

 Another phenomenon – multiple terraces is also common in nature. Small-size breach in the crust and release of a tiny amount of molten material results in the level decrease with subsequent self-repairing and crust solidification in the new position. A multiple terrace structure, as the ones shown in the Unit 1 (Fig. 6) and at the sea bottom (Fig. 7), are usually formed by cycling of the “release-repair” mechanism several times.

**Table 1:** A comparison of images from the 1F observations with natural lava flows.

1F debris in the Unit 1	Natural lava flows
	
Fig. 2. The solid crust formed at an elevation where corium concrete interaction lava stably attached to the walls and melted concrete structures in the PCV of the Unit 1. Source: TEPCO^23^.	Fig. 3. Solidified lava during eruption at Kīlauea with surface similar to debris of the 1F Unit 1. Source: Youtube^24^
	
Fig. 4. Smooth surface of the 1F Unit 1 molten silicate-based terrace debris. Source: IRID and TEPCO^25^.	Fig. 5. Burst of submarine pillow lava with spilled material and stabilized hollow crust. Source: NOAA^26^.
	
Fig. 6. Multiple terrace structures at the 1F Unit 1. Source: IRID and TEPCO^25^	Fig. 7. Terrace structures after the submarine lava ejection at Vailulu’u. Source: NOAA^26^

## Discussion

The previous section highlighted many similarities with natural lava propagation. Therefore, it is proposed that the formation and spreading of volcanic lava above the earth surface and MCCI product in the 1F Unit 1 pedestal had similar mechanism and similar temperature conditions. The objection that the debris could be (U, Zr)O_2_ hot corium can be rebutted. Visually, spreading of molten (U, Zr)O_2_ corium is similar to volcanic lavas. However, one has to match visual features and the consequences of the interaction. Considering the fact that rebars have survived, currently the logical assumption can be summarized as follows. When volcanic lava attacks metallic structures they survive, when corium attacks, temperature is much higher – then they melt. Metal fence at Hawaii and metal rebars of 1F Unit 1 survived, thus in the pedestal of the 1F Unit 1 is not purely corium, but already a product of MCCI reaction. Conditions for melting of concrete have to be the same or nearly the same as for volcanic lava, i.e. temperatures around 1200–1250 °C. Corium would be entirely frozen at this temperature. That is why it was assumed that in the pedestal of 1F Unit 1 we see a lot of MCCI product, not the corium itself. Furthermore, it can be assumed that 1F lava has similar nature with volcanic lava. It may be a silicate-based lava in which a certain amount of the Unit 1 molten corium can be dissolved.

If it is true that the 1F MCCI lava near the pedestal opening is silicate-based material, then temperature conditions for stabilizing the crust should be similar. If crust of volcanic lava forms under subaerial condition of summer weather at Hawaii, then roughly, the same temperature is expected in the pedestal bottom of the Unit 1 right before the MCCI and relocation of molten lava.

The notion above contradicts the calculation of Sevon^27^ who predicted that the drywell temperature at the time of relocation of the debris should be around 160 °C. The calculation was based on assumption of leak of steam directly to the drywell. Drywell temperature in the calculation is averaged over the entire large volume. There is no difference at the top-middle-bottom areas. However, a small volume near the assumed leak was called “drywell hot” and it has become the heat source for the entire volume. At the same time, real temperature distribution is always a gradient, and surely, before debris relocation the coldest area is the pedestal bottom. There is no doubt that the actual temperature at the bottom was much lower than 160 °C at the moment of debris relocation, while at the top of the drywell it could be higher. In average, it yields 160 °C, but it would not represent the situation in the pedestal bottom. Probably, this contradiction cannot be resolved easily now and should be further investigated by checking the possibility to form crust at higher ambient temperatures and by more detailed calculation of the drywell volume which is separated into bottom, middle, top drywell parts. One has to admit, that the conditions in the PCV pedestal bottom resulted in the fact that the crust has been formed by heat removal at the interface, due to convection and radiation losses, while below the crust 1F MCCI lava remained liquid and continued to degrade the concrete until it was completely lost. For a further work, it is required to perform some specifically dedicated numerical simulations, which could probably serve as a first technical estimation of the temperature distribution inside the PCV at the moment of crust formation and lava spreading.

Smooth lava surface appearance, implies that the 1F Unit 1 lava flow in the pedestal area was slow with relatively low viscosity. In volcanology, the ‘a’ā lava type implies a viscosity in the range from 1.3 × 10^5^ to 3.3 × 10^7^ Pa∙s with rough surface and the smooth pahoehoe lava is less viscous from 5 × 10^3^ to 5 × 10^4^ Pa∙s^28^. However, in general both types of lava consist of the same material. The behaviour of multi-layered lava flows is complex and the regime of lava flow decides more than the viscosity of the lava itself. In turn, surface topology has large influence on the flow regime. In the Tolbachik volcano eruption, the lava flows transited from pahoehoe to ‘a’ā on a steep flow. However, with time it had changed from ‘a’ā to pahoehoe by creation of the network of lava tubes^28^. The tube wall insulated lava and provided more space for more volume of hot lava, which brought more heat, created a large field of lava and thus decreases the viscosity of the lava flow in tubes^28^. In 1F, the role of the lava tube walls has been successfully taken over by the pedestal walls. Large volume of pahoehoe-like 1F MCCI lava created a smooth surface, similar to the case of the volcanic lava flows. The average viscosity of the 1F MCCI lava flow thus can be roughly estimated to be in the range of 10^3^ to 10^4^ Pa∙s because only this kind of viscosity allows the complex behaviour of spreading with smooth surface, together with smooth crust formation and possibility of piling-up. Viscosity values of 10^3^ Pa∙s was confirmed experimentally for 90% SiO_2_ and 10% corium mixture at 1650°C^29^. Thus, 1F Unit 1 PCV terraced debris would be linked rather to lower viscosity pahoehoe lava then to ‘a’ā lava or corium.

Same as described in Belousov and Belousova^28^ the 1F Unit 1 lava was able to pile-up, while the inner part was liquid. The less viscous layer temperature should be around 1250–1400 °C to remain liquid under the crust completely, but to let the reinforcement bars survive. The characteristic surface structure of rebars is well recognized on TEPCO’s videos. The Osaka University researchers took thinner rebar and oxidized in air for 8 h at 1200 °C. As confirmed by the experiments^13^ though partly degraded, the surface structure can be recognized same as on TEPCO’s videos. Considering different rebar degradation conditions because in the 1F they were covered by the liquid lava, the conditions for rebars survival could be less severe in the case of 1F rather than high-temperature oxidation in air. It is not a surprise that the rebars of 1F survived in better state than those after long-time high-temperature oxidation in air.

There is no doubt that beneath the crust the lava was liquid. Only if the inner part was liquid, it could clean the rebars and penetrate through metallic reinforcement structures without destroying them. And only liquid material can change the level quickly, that there is not enough time for cooling and attaching to the rebars. Thus, it can be assumed that when the crust was suddenly broken, the liquid material quickly spilled to outside pedestal area and the overall level near the pedestal opening had dropped. In the central areas, the crust followed the new level of lava. However, in the peripheral regions the “terrace” was anchored to the walls and reinforcement steel bars. The terraces were observed mostly outside of the pedestal. It means that only outside of the pedestal the temperature gradient was enough to stabilize the crust.

Let us now examine the composition of crust. Careful consideration of facts proved that the 1F crust could not be made of solely UO_2_ corium debris; if it did, the temperature of the underlying liquid (or even partly liquefied) material would exceed the steel melting temperature, resulting in failure of the steel reinforcement bars. Nonetheless, it is evident that UO_2_ had intermixed and dissolved within the molten concrete, contributing thermal energy that facilitated the lava’s penetration through the concrete wall. The question remains: in what location the interaction between corium, metallic debris and concrete occur.

**Fig. 8:**
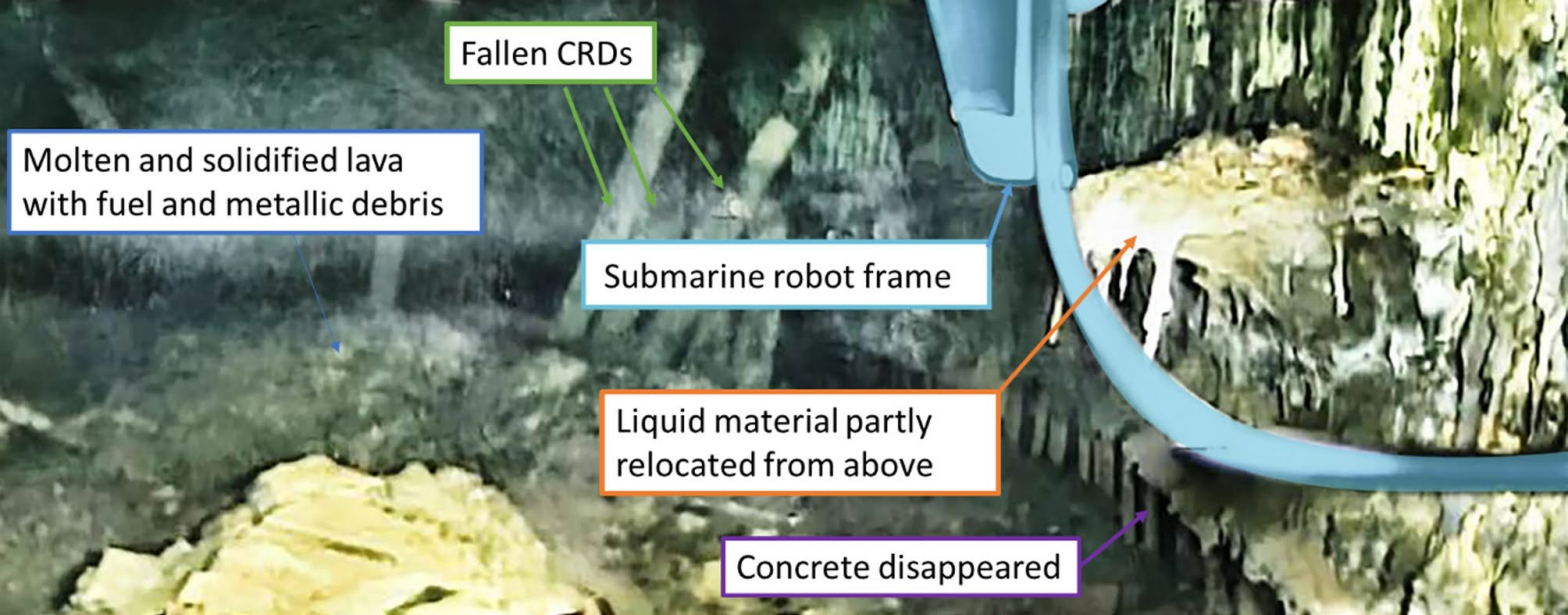
Evidence that a part of the molten material relocated from the top part of the pedestal where the unconventional MCCI could happen. Source: TEPCO^2^.

 At a Nuclear Regulation Authority meeting it was mentioned that if all of the core components would melt, the height of the molten pool in the pedestal would be only around 1 m high^30^. However, the observed crust terrace remains were detected at the height of around 1.5 m as estimated by the total height of the pedestal opening (1.9 m high). A volume increase of this kind would never be possible by classic MCCI at the floor. One of the key suggestions of this paper is that the first time MCCI in the Unit 1 might have happened at the upper part of the pedestal not on the floor, as everybody usually understand it. An evidence for this was published by TEPCO in the video report^2^. A snapshot from the video can be seen in the Fig. 8.

In the pedestal inner wall image, there were many rivulets coming from a mass of previously molten material attached to the wall showed relocation of molten materials from above. MCCI may have started first above this point. These rivulets cannot be made of liquid and then solidified corium, because the liquid corium has viscosity lower than that of water^31^ and then it quickly solidifies providing less possibility for the thin rivulet flow. Thus, the rivulets are considered to be already 1F MCCI lava product.

**Fig. 9:**
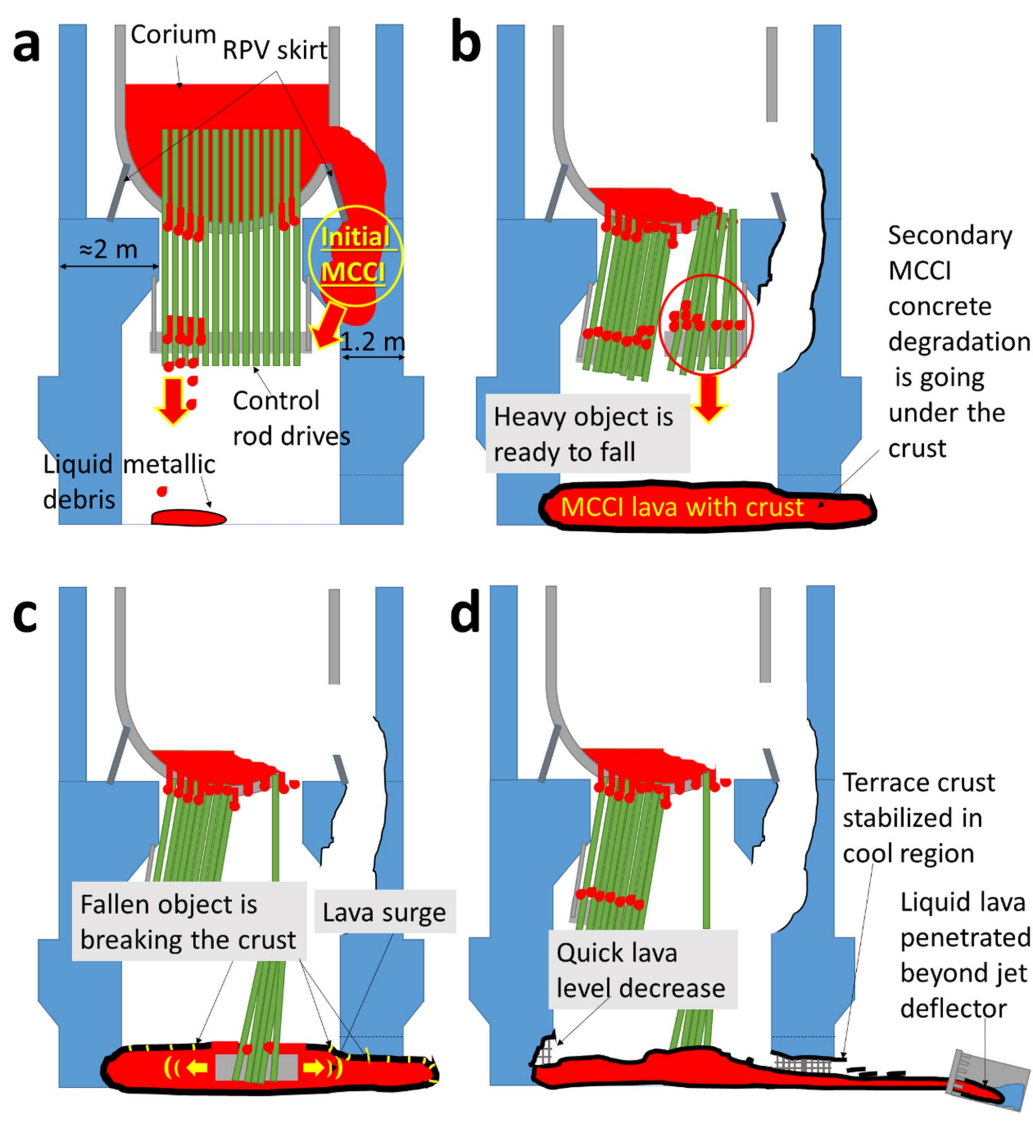
Schematic representation of a – proposed new place of MCCI; b – relocation of MCCI product via the lava tube, solidification at the pedestal floor with first crust formation in a cold PCV environment, c – falling object causes braking of the crust and a wave of lava release out of pedestal opening; d – fast release of lava caused terrace-like remains outside of the pedestal, some material penetrated beyond the jet deflector and contacted with cooling water in the suppression chamber.

As a second point of discussion, this paper proposes a sequence of the corium formation and relocation in 1F Unit 1, as illustrated in the Fig. 9. This sequence elucidates the upper MCCI point, the first crust formation and subsequent breaking of crust. The author posits that for this scenario to occur, corium must have melted through the RPV above the RPV skirt and subsequently become trapped between the RPV skirt and a concrete structure (see Fig. 9a). This idea can be supported by numerous tests showing the damage of RPV lower head by the “focusing effect” at around 70–80° (0° is RPV downward direction). A comprehensive description of this and the other RPV failure modes can be found for example in B.R. Sehgal’s book Chapter II^32^. The focusing effect happens because of melt stratification into oxide and metal layers when metallic layer becomes over the debris and transports all heat directly to the local area in the RPV wall. Large heat flux damages it by melting. Then, the hole ablates by the hot corium and in general, this place becomes major breach.

It is difficult to think of a more suitable location for the initial MCCI as near such place where major breach had occurred. In the 1F Unit 1 BWR reactor case, approximately 2-meter-thick concrete in that area facilitated production of a large amount of silicate-based melt right above the pedestal opening. The interaction between the corium and the concrete led to a substantial additional mass of MCCI lava material, which was enough to account for 50 cm of extra material that could explain observed increase in the height of the molten debris from 1 m to 1.5 m providing there was no strong floor melting.

Up to date it is still difficult to confirm where the major melt relocation from RPV had occurred. The place of assumed upper MCCI is still not sufficiently investigated by the robots. This information is crucial for understanding of the accident progression and author’s personal hope that it will be investigated in the near future. When we understand the exact place of major RPV breach, the severe accident community would have a breakthrough in understanding of the accident progression of the 1F Unit 1.

According to the second scenario of CRD nozzle and instrumentation tube damage^32^ indeed, some part of the RPV in-vessel corium melt may have reached the pedestal bottom without the occurrence of MCCI by seeping through the RPV lower head, particularly near the CRD nozzles. Small-scale leaking is likely to start before the major breach. Later, large-scale melt release and small-scale leaking can develop in parallel. After major part of corium has relocated, there is no powerful heat source in RPV, thus the remaining melt in RPV should be able to freeze where it was. In addition, there will be no internal RPV pressure, which helped ejecting the melt through small breaches. If the main role were only due to the RPV lower head breach without MCCI, one would not anticipate the increase of the debris amount. Therefore, the contribution of relocation through the CRD nozzles seems minimal, while upper MCCI role is maximal for the Unit 1. The process of UO_2_ and metallic debris relocation, together with the initial MCCI location, are illustrated in the Fig. 9a.

In the Fig. 9b, both the vessel and the pedestal wall exhibit the signs of degradation, and major melt relocation has been accomplished. It had resulted in a molten lava mass (MCCI product) that overflowed the pedestal and slightly extended outwards. However, due to temperature gradient the flow stopped and material covered by a solid crust. In the absence of external influences, further development would normally halt, as it happens in nature. Nonetheless, one more critical factor should be considered in case of the 1F Unit 1. The mechanism of submarine crust stabilization indicates that a crust stabilization should be accompanied by rapid liquid outflow with rapid cooling.

What could break the crust and cause the liquid lava to flow out of the pedestal? An example of this phenomenon is shown in the Fig. 9c. Apparently, a set of CRD that were actually observed on the Unit 1 investigation video^2^ collapsed together with the part of the CRD supporting structures and RPV corium accumulated on them (that CRDs also visible in the Fig. 8).The large weight of this object could disrupt the crust, triggering a surge of lava. This surge would lead to a rapid release of lava from the pedestal opening, extending towards the opposite jet deflector. Fast decrease in the lava level combined with relatively cooler environment outside of the pedestal resulted in crust stabilization (Fig. 9d). Furthermore, the jet deflector only impeded the lava flow but had not stopped it. Similar to the observations in the Fig. 3, while lava contacts metallic surface, it remains in a liquid state within the bulk much like in volcanic lava tubes. Local crust damage can occur, allowing lava flow to continue beyond the jet deflector, which at the end contributed to formation of the multiple terraces as illustrated in the Fig. 6.

It is assumed that the lava advanced beyond the jet deflector until it reached water within the suppression chamber (Fig. 9d). Largely this mechanism is similar to natural lava flows at Hawaii with subsequent lava discharge to the ocean. In the context of the 1F Unit 1 assumed scenario, such contact of corium and fuel containing silicate lava with coolant water can be considered as a potential fuel-coolant interaction (FCI). It inevitably leads to transport of actinides dissolved in lava into the cooling water of the suppression chamber. The first evidence of the FCI for the Unit 1 has been already published^33^. Similar situation has been already reported for the Unit 2^34^ and the Unit 3^35^. Typical corium particles containing U-Zr-O as main elements, were observed attached to agglomerates consisted of Al, Na, Si, Fe, O elements. If such particles were found in the Unit 2 where the possibility of the MCCI was considered low, for the Unit 1 it can be assumed, that the concentration of the same style particles in the water of suppression chamber might be much higher. Some particles could have originated from agglomeration of smaller fragments of quenched lava. For some of them the mechanism formation could be chemical precipitation of the leached actinides from solution onto micrometre-scale fragments of broken lava. Indeed, recent work has confirmed relatively high U, Np, Pu, Am concentration in the suppression chamber stagnant water of Units 1-3^33,36^. Both cases of actinides bearing particle formation mechanisms in the 1F Unit 1 suppression chamber are not excluded.

## Conclusion

An interdisciplinary approach from volcanology to severe nuclear accident was applied to describe and interpret the unconventional MCCI discovered in 1F Unit 1. The analysis suggests that MCCI during severe accidents can happen in unpredictable place, which should be taken into account in all future accident analyses. The following conclusions can be derived from the input image analysis and subsequent discussions:

- The paper provided insight on 1F Unit 1 lava debris behaviour from volcanology perspective, which is a field of science distinct from that of severe accidents, once again proving that to enhance the understanding of the accident’s progression at 1F Unit 1 an interdisciplinary collaboration is beneficial.
- A novel understanding of MCCI process has been proposed and it should be taken into account when considering severe accident analysis of the 1F Unit 1; Initially RPV breach occur above the RPV skirt, then major melt relocation starts MCCI in an atypical location at the top of the pedestal wall, followed by conventional MCCI occurring on the pedestal floor.
- The process of formation of the terrace-like structures observed outside of the pedestal in 1F Unit 1 was proposed based on comparisons with natural lava flow behaviour, in particular that of the submarine pillow lava and volcanic lava tubes.
- The temperature gradient associated with natural lava flows and the image of destruction it had caused, allows suggesting limits in temperature within the pedestal under the crust may have been in a range of 1250–1400 °C. Outside of the pedestal, where stable lava crust had formed and naturally anchored on the cooler surfaces without destroying those, the temperatures of lava outer surface were unlikely to exceed 800 °C.
- Because of fuel-coolant interaction, concentration of U-bearing particles in stagnant water of the suppression chamber can be high.

For a more comprehensive accident analysis, it would be beneficial to engage experts from the field of volcanology. It helps interpreting the visual consequence of the accident and re-establish the accident progression by taking into account for different material properties of corium and MCCI lava product. In the modelling of liquid corium flow using equations derived from lava spreading, some particular success has been achieved by interdisciplinary approach of experts from volcanology and severe accidents. Such work should be continued. Probably existing models of lava flow and crust formation could be adapted and refined to incorporate the specific MCCI features of the 1F, such as braking crust or the impact of a falling heavy object to the lava molten pool.

## Data Availability

The author declares that the data supporting the findings of this study are available within the paper from direct links and additional data can be found in repositories: https://www.tepco.co.jp/library/movie/https://oceanexplorer.noaa.gov/explorations/05vailuluu/.

## Abbreviations

1F: Fukushima Dai-Ichi Nuclear Power Plant
CRD: Control Rod Drive
FCI: Fuel–Coolant Interaction
IRID: International Research Institute for Nuclear Decommissioning
MCCI: Molten Corium–Concrete Interaction
NEA: Nuclear Energy Agency
NOAA: National Oceanic and Atmospheric Administration
OECD: Organisation for Economic Co-Operation and Development
PCV: Primary Containment Vessel
RPV: Reactor Pressure Vessel
TEPCO: Tokyo Electric Power Company
USGS: United States Geological Survey
